# Application of immuno- and affinity labeling with fluorescent dyes to in-resin CLEM of Epon-embedded cells

**DOI:** 10.1016/j.heliyon.2023.e17394

**Published:** 2023-06-17

**Authors:** Isei Tanida, Junji Yamaguchi, Chigure Suzuki, Soichiro Kakuta, Yasuo Uchiyama

**Affiliations:** a Department of Cellular and Molecular Neuropathology, Research Institute for Diseases of Old Age, Juntendo University Graduate School of Medicine, Tokyo, Japan; b Laboratory of Morphology and Image Analysis, Biomedical Research Core Facilities, Juntendo University Graduate School of Medicine, Tokyo, Japan; c Department of Cellular and Molecular Pharmacology, Juntendo University Graduate School of Medicine, Bunkyo-Ku, Tokyo, Japan; d Center for Diversity and Inclusion, Juntendo University School of Medicine, Bunkyo-Ku, Tokyo, Japan

**Keywords:** Fluorescent dye, Osmium tetroxide, Correlative light and electron microscopy, Epoxy resin

## Abstract

In-resin CLEM (Correlative Light and Electron Microscopy) of Epon-embedded cells involves correlating fluorescence microscopy with electron microscopy in the same Epon-embedded ultrathin section. This method offers the advantage of high positional accuracy compared to standard CLEM. However, it requires the expression of recombinant proteins. In order to detect the localization of endogenous target(s) and their localized ultrastructures of Epon-embedded samples using in-resin CLEM, we investigated whether immunological and affinity-labeling using fluorescent dyes applied to in-resin CLEM of Epon-embedded cells. The orange fluorescent (λ_em_ ∼550 nm) and far-red (λ_em_ ∼650 nm) fluorescent dyes examined maintained a sufficient level of fluorescent intensity after staining with osmium tetroxide and subsequent dehydration treatment with ethanol. Immunological in-resin CLEM of mitochondria and the Golgi apparatus was achieved using anti-TOM20, anti-GM130 antibodies, and fluorescent dyes. Two-color in-resin CLEM revealed that wheat germ agglutinin-puncta showed the ultrastructures of multivesicular body-like structures. Finally, taking the advantage of high positional accuracy, volume in-resin CLEM of mitochondria in the semi-thin section (2 μm thick) of Epon-embedded cells was performed by focused ion beam scanning electron microscopy. These results suggested that the application of immunological reaction and affinity-labeling with fluorescent dyes to in-resin CLEM of Epon-embedded cells is suitable for analyzing the localization of endogenous targets and their ultrastructures by scanning and transmission electron microscopy.

## 1 Introduction

In-resin CLEM, or correlative light and electron microscopy using polymer-resin-embedded ultrathin sections, provides a method for accurately correlating fluorescence microscopy with electron microscopy. This technique offers higher positional accuracy compared to standard CLEM procedures [[1], [2], [3]]. In standard CLEM [4], fluorescent signals in the cells are typically acquired prior to embedding samples in epoxy or other types of polymer-resins. The cells are then chemically fixed using a paraformaldehyde and glutaraldehyde mixture, stained with osmium tetroxide, dehydrated using a graded series of ethanol, and finally embedded in resins. Following the preparation of ultrathin sections (50–100 nm) from resin-embedded samples, electron microscopic images are acquired. It is important to note that during the preparation process, the chemical and physical distortions can occur, which may reduce the correlation between fluorescence and electron microscope images. Additionally, there is a discrepancy in the Z-axis resolution between fluorescent (typically several μm thick) and electron microscopic images (usually 50–100 nm thick).

In order to address the limitations of standard CLEM, the technique of in-resin CLEM has been developed [[5], [6], [7], [8], [9]]. In-resin CLEM aims to capture both fluorescence and electron microscopic images from the same thin section of resin-embedded specimens. However, it is important to note that there are specific limitations associated with in-resin CLEM, particularly for embedding samples in ‘Epon’ resins and osmium tetroxide staining. Embedding samples in Epon resins is a reliable technique for preserving intracellular ultrastructures in cells and tissues for electron microscopy. Osmium tetroxide staining plays a crucial role in the visualization of membranous structures in electron microscopy [10]. However, Epon resins exhibit autofluorescence, which can interfere with fluorescence imaging. Additionally, osmium tetroxide treatment can lead to a reduction in the fluorescent intensity of various fluorescent proteins and fluorophores [6,11]. However, certain fluorescent proteins, such as mEosEM, mEosEM-E, mWasabi, CoGFP variant 0, mCherry2, mKate2, and mScarlet-H, have been found to be resistant to osmium tetroxide staining [[7], [8], [9],^11^,^12^]. This characteristic allows for successful multicolor in-resin CLEM of Epon-embedded cells using these green and red fluorescent proteins has been achieved [9,13]. Two color in-resin CLEM with self-labeling tags (SNAP- and CLIP-tags) to label insulin-secretory granules and autophagosome-marker LC3 has been successfully performed in thin sections (300 nm thick) of Epon-embedded cells [14]. Recently, a novel approach in in-resin CLEM has emerged, which proximity labeling using a biotin-ligase, miniTurbo. This technique has been shown to provide higher fluorescent intensity and greater stability in Epon-embedded cells compared to in-resin CLEM using fluorescent proteins [13,15]. However, these techniques require molecular engineering to express the recombinant protein(s) in the cells and tissues to perform in-resin CLEM. In addition, nonspecific labeling protocols for endogenous targets (nuclei, cell membrane, cytoskeleton, lipophilic domains, entire cells and tissues) have been reported for in-resin CLEM. However, these techniques are only suitable for a very limited number of targets and cannot be used to analyze specific endogenous proteins using in-resin CLEM [16]. Regarding in-resin CLEM for endogenous protein targets, in-resin correlative super-resolution fluorescent and electron microscopy of immunolabeled mitochondria and cytoskeleton in the epoxy resin-embedded samples has been challenged [17]. In this case, fluorescent images correlated well with electron microscopic images, but ultrastructures of membranous compartments including mitochondria were poorly preserved [17]. Therefore, a new method for in-resin CLEM of endogenous target(s) in Epon-embedded cells has been long awaited.

In this study, we applied immunological and affinity-labeling with fluorescent dyes for in-resin CLEM, and achieved in-resin CLEM of endogenous targets in the Epon-embedded samples using both scanning and transmission electron microscopy. Furthermore, taking the advantage of high positional accuracy in Epon-embedded cells, we performed volume in-resin CLEM of mitochondria via FIB-SEM **(**focused ion beam scanning electron microscopy) detecting endogenous mitochondrial protein immunologically.

## 2 Results

### 2.1 Fluorescence of orange and far-red fluorescent dyes was preserved after osmium tetroxide staining and dehydration with ethanol

**Fig. 1:**
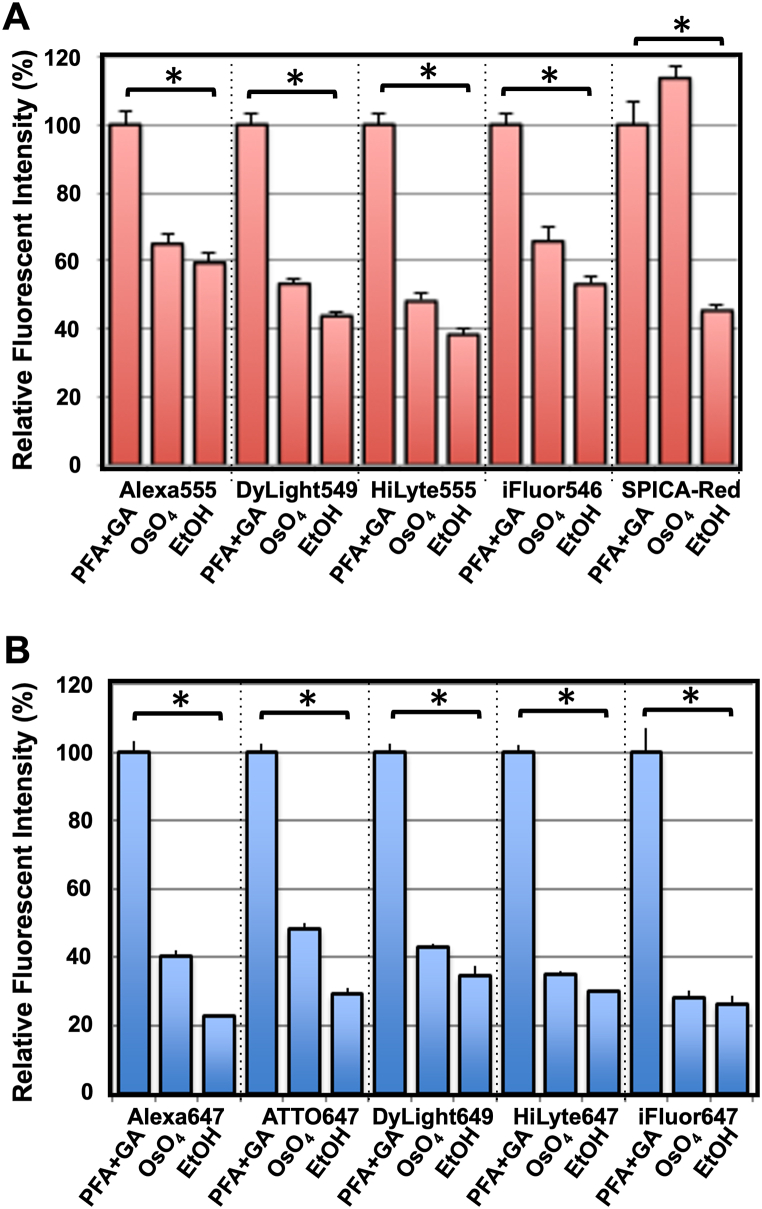
**Fluorescent intensity of fluorescent dyes in the HeLa cells was partially preserved after osmium tetroxide staining and dehydration with ethanol following the fixation with glutaraldehyde.** HeLa cells were fixed in the mixture of paraformaldehyde and glutaraldehyde. After permeabilization, cells were incubated with biotinylated WGA and fluorescent dye-conjugated streptavidin. Cells were fixed in the mixture of paraformaldehyde and glutaraldehyde (**PFA + GA**), stained with osmium tetroxide (**OsO**_**4**_), and dehydrated with ethanol (**EtOH**). After detecting the fluorescent signals in the cells, relative fluorescent intensity was calculated when the intensity of each fluorescent dye in **PFA + GA** was regarded as 100% (n > 2000, p < 0.01). Asterisks indicate p < 0.01 for the “**PFA + GA**” and “**EtOH**” groups when tested for significant differences by student t-test.

Autofluorescence of 100 nm thin section of Epon-embedded cells is negligible during in-resin CLEM when fluorescence intensity derived from the fluorescence-labeled cells and/or organelles is high enough [7,9,15]. We investigated the resistance of orange fluorescent dyes (λ_em_ ∼550 nm) against osmium tetroxide and ethanol when cells were labeled with biotin-conjugated WGA and fluorescent dye-conjugated streptavidin. HeLa cells were fixed with 4% paraformaldehyde and 0.25% glutaraldehyde. After permeabilization of the cells with 50 μg/ml digitonin and 0.02% Triton X-100, cells were incubated with biotinylated WGA and fluorescent dye (Alexa Fluor 555, DyLight 549, HiLyte 555, iFluor549, and SPICA Red)-conjugated streptavidin. Thereafter, cells were fixed with 2% paraformaldehyde and 2.5% glutaraldehyde, and fluorescent images were obtained with a BZ-X810 fluorescence microscope (Fig. 1A, PFA + GA). Next, cells were postfixed with 2% osmium tetroxide at 4 °C for 30 min (Fig. 1A, OsO_4_), and dehydrated with 100% ethanol (Fig. 1A, EtOH). WGA-positive fluorescence of each fluorescent dye in the cells was observed well after the treatment with osmium tetroxide and ethanol. Quantification of the fluorescence intensity of WGA-positive signals in the cells revealed that about 40% of the fluorescence was preserved after dehydration with ethanol following osmium tetroxide staining.

We next focused on far-red fluorescent dyes. When far-red fluorescent dyes (λ_em_ ∼650 nm) (Alexa Fluor 647, ATTO 647, DyLight 649, HiLyte 647, and iFluor 647) were employed instead of orange fluorescent dyes, the fluorescence of WGA-positive signal was observed well, albeit attenuated, after dehydration with ethanol following osmium tetroxide staining (Fig. 1B). Quantification of fluorescent intensity indicated that about 20∼30% of the fluorescence was preserved after dehydration with ethanol (Fig. 1B, PFA + GA vs EtOH). These results suggested that these fluorescent dyes were potential candidates for the application of affinity-labeling with fluorescent dye for in-resin CLEM.

### 2.2 Immunological reactions were applied to in-resin CLEM of mitochondria and the Golgi apparatus in Epon-embedded cells

**Fig. 2:**
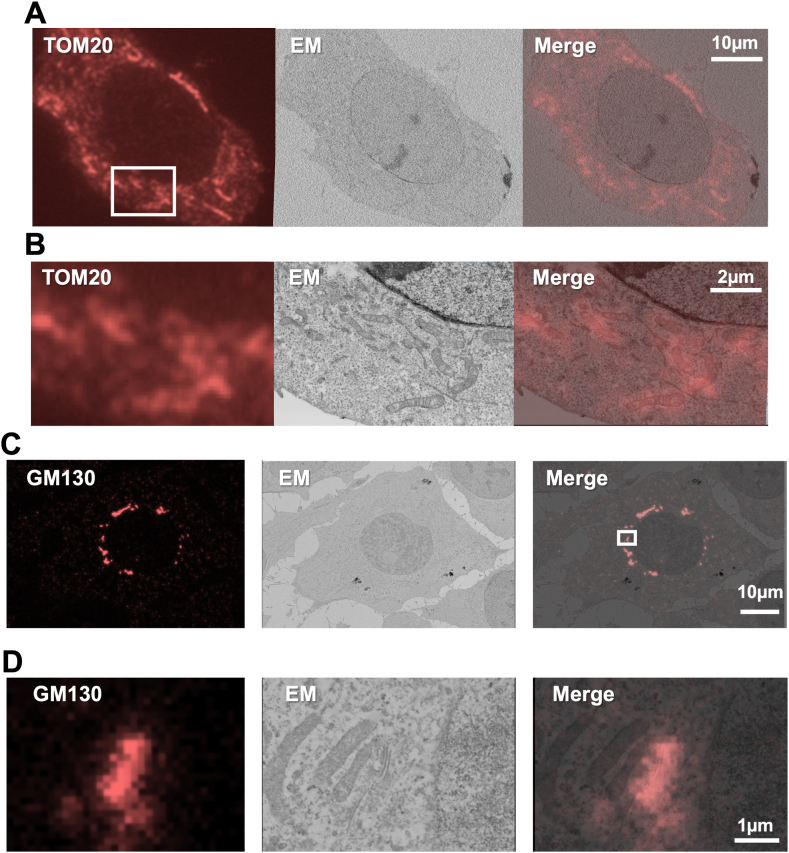
**Immunological reactions with orange fluorescent dye were applied for in-resin CLEM of mitochondria and the Golgi apparatus in the Epon-embedded cells.** After fixation in the presence of glutaraldehyde and permeabilization, HeLa cells were incubated with rabbit polyclonal anti-**TOM20** (**A** & **B**) and mouse monoclonal anti-**GM130** (**C** & **D**) antibodies. After washing the cells, cells were incubated with biotinylated anti-rabbit IgGs (**A** & **B**) and anti-mouse IgGs (**C** & **D**), and further incubated HiLyte 555-(**A** & **B**) and iFluor 546-(**C** & **D**) conjugated streptavidin. After osmium tetroxide-staining and dehydration with ethanol, cells were embedded in Epon-resins. After preparation of 100 nm thin sections, fluorescence in the section was observed using a BZ-X810 fluorescence microscope (CCD monochrome camera, Nikon CFI Plan Apochromat Lambda 100x Oil lens, gain +16 dB) using a TRITC filter set (excitation: 520–570 nm, dichroic mirror: 565 nm long pass, emission: 535–675 nm) (**A** & **B**) or a Nikon A1RHD25 confocal laser-scanning microscope (Nikon CFI Plan Apochromat Lambda 60x lens) (**C** & **D**) (**FM**). Electron microscopic images (**EM**) were obtained with a Regulus 8240 scanning electron microscope (a backscattered electron detector at a voltage of 2.0 kV). The “**merge**” is the merged image of the fluorescence (**FM**) and electron microscopic images (**EM**).

We next investigated whether it is possible that immunological reaction and fluorescent dye using an antibody for in-resin CLEM of Epon-embedded cells. HeLa cells were fixed with 4% paraformaldehyde and 0.25% glutaraldehyde, and permeabilized with 50 μg/ml digitonin and 0.02% Triton X-100. After blocking the cells with 1% BSA, cells were incubated with rabbit polyclonal anti-TOM20 antibody in the presence of 0.1% BSA. Following the incubation with biotinylated goat anti-rabbit IgG antibody, cells were incubated in a buffer with HiLyte 555-conjugated streptavidin. After osmium tetroxide staining and dehydration with ethanol, cells were embedded in Epon resins. After the preparation of 100 nm thin sections of Epon-embedded cells, fluorescent images in the section were obtained with a BZ-X810 fluorescence microscope (Fig. 2A and B, FM). Fluorescent signals of anti-TOM20 antibody were obtained well in the thin section of Epon-embedded cells and showed mitochondria-like localization patterns. Ultrastructures of membrane structures including mitochondria were well-preserved when the same sections were investigated by a Helios NanoLab 660 SEM (Fig. 2B, EM). Fluorescent signals derived from anti-TOM20 antibody were well correlated with mitochondrial structures in the electron microscopic images (Fig. 2A and B, merge). These results suggested that in-resin CLEM of mitochondria in the Epon-embedded cells using immunological reactions with a HiLyte 555 fluorescent dye was achieved.

We next investigated whether this application of immunological reactions using an antibody for in-resin CLEM of the Golgi apparatus in the Epon-embedded cells. We employed for anti-GM130 (a marker of the Golgi apparatus) antibody and iFluor 546-conjugate streptavidin instead of anti-TOM20 antibody and HiLyte 555-comjugated streptavidin, respectively. After fixation of the cells in the presence of glutaraldehyde as described above, cells were immunologically reacted with mouse monoclonal anti-GM130 antibody, biotinylated secondary anti-mouse IgGs antibody, and iFluor 546-conjugated streptavidin. After the preparation of the Epon-embedded cells, 100 nm thin sections were prepared. Fluorescent images in the section were obtained with a Nikon A1RHD25 confocal laser-scanning microscope (Fig. 2C and D, FM). Fluorescent signals derived from the anti-GM130 antibody were observed in the 100 nm thin sections, showing the Golgi-like pattern. Electron microscopic images of the same area in the section were obtained by a Regulus 8240 scanning electron microscope (Fig. 2C and D, EM). Electron microscopy showed that ultrastructures of the Golgi apparatus and Golgi-related vesicles were observed in the fluorescent positive area (Fig. 2D, EM). These results suggested that in-resin CLEM of the Golgi in the Epon-embedded cells using immunological reactions and fluorescent dyes was achieved when the antibody was reacted with endogenous target in the fixed cells.

### 2.3 WGA-positive vesicles were characterized as multivesicular body-like structures by the application of affinity-based reactions to two-color in-resin CLEM of Epon-embedded cells

WGA binds to N-acetylglucosamine and N-acetylneuraminic acid (sialic acid) residues and is considered to label the cellular membrane and post-Golgi structures of mammalian cells. Intracellular WGA-positive signals were often observed as puncta in the 100 nm thin section of the Epon-embedded HeLa cells by a fluorescence microscope. Taking the advantage of high positional accuracy in the correlation between fluorescence and electron microscopic images of in-resin CLEM of Epon-embedded samples, we investigated the ultrastructures of intracellular WGA-positive signals in the Epon-embedded cells by in-resin CLEM. To identify the WGA-positive signals, we focused on mitochondria as an intracellular positional and ultrastructural marker available for both fluorescence and electron microscopy. The reason for this is that in addition to fluorescence indicating the presence of mitochondria by anti-TOM20 antibody distributed intracellularly, the ultrastructure of mitochondria by electron microscopy is distinctive (Fig. 2B). As far as two-color in-resin CLEM using anti-TOM20 antibody and WGA is achieved, it will be useful as an internal position and morphological marker to identify the WGA-positive ultrastructures and signals.

**Fig. 3:**
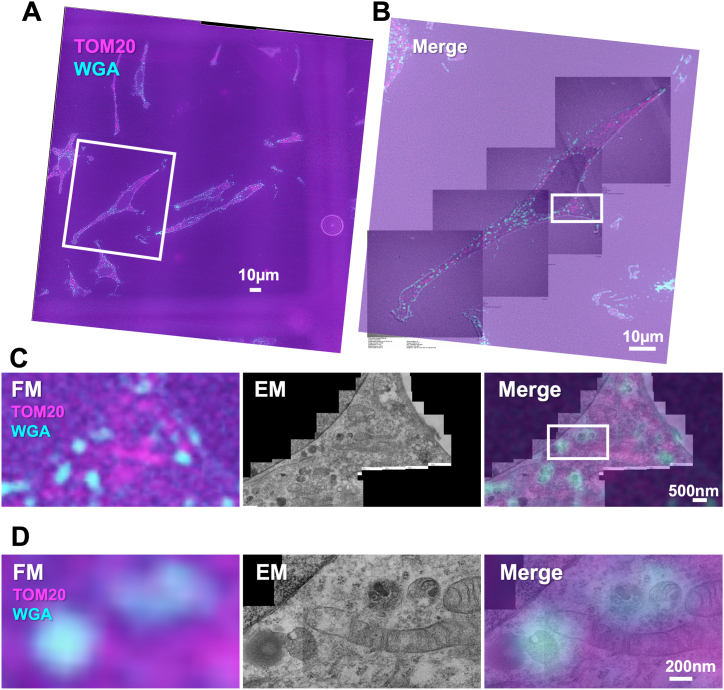
**Two-color in-resin CLEM of intracellular organelles in Epon-embedded cells was performed using orange and far-red fluorescent dyes.** After staining **TOM20** using SPICA-red streptavidin as described in Fig. 2, cells were further incubated with Alexa Fluor 647-conjugated **WGA**, stained with osmium tetroxide, and dehydrated with ethanol. After Epon-embedding of the cells, 100 nm thin sections were prepared and collected on TEM grids. Fluorescent images in the section were observed using a BZ-X810 fluorescence microscope (CCD monochrome camera, Nikon CFI Plan Apochromat Lambda 100x Oil lens, gain +16 dB) using TRITC (excitation: 520–570 nm, dichroic mirror: 565 nm long pass, emission: 535–675 nm for orange fluorescent dye) and Cy5 filter sets (excitation: 560–680 nm, dichroic mirror: 660 nm long pass, emission: 625–775 nm for far-red fluorescent dye) (**FM**). Electron microscopic images (**EM**) were obtained with an HT7700 transmission electron microscope. The “**merge**” is the merged image of the fluorescence (**FM**) and electron microscopic images (**EM**). The images in **B**, **C**, and **D** are high-magnified images of the area with a white square surrounding fluorescent image in **A**, **B**, and **C**, respectively.

To perform two-color in-resin CLEM, HeLa cells were fixed in the presence of glutaraldehyde, and intracellular mitochondria were immunologically detected with the anti-TOM20 antibody with SPICA red-conjugated streptavidin. Cells were further incubated with Alexa Fluor 647-conjugated WGA since Alexa Fluor 647 was also resistant to osmium tetroxide staining and dehydration with ethanol (Fig. 1). After incubation with osmium tetroxide and dehydration with a graded series of ethanol, cells were embedded in the Epon resins. After the preparation of a 100 nm thin section of the Epon-embedded cells on a TEM grid, fluorescent signals derived from WGA and TOM20 were obtained by a BZ-X810 fluorescence microscope (Fig. 3, FM). TOM20-positive signals were detected as mitochondria-like structural patterns in the thin section on the TEM grid, while WGA-positive signals were also detected well as intracellular puncta in addition to the cellular membrane. Intracellular ultrastructures of the cells in the same area of the same section were analyzed by an HT7700 TEM (Fig. 3, EM). Under these conditions, ultrastructures of membrane organelles including mitochondria were well preserved. Correlation of fluorescence with electron microscopic images of ultrathin sections in the Epon-embedded cells indicated that TOM20-positive signals in the fluorescent images were well correlated with the ultrastructures of mitochondria in the electron microscopic images. According to the correlation with high positional accuracy, we identified the ultrastructures of WGA-positive signals. Higher magnified electron microscopic images revealed that WGA-positive structures seemed to be late endosome-like structures such as multivesicular bodies. These results indicated that two-color in-resin CLEM of endogenous membrane structures in the Epon-embedded samples was able to be achieved using immunological and affinity-based reactions with two-color fluorescent dyes, and that intracellular WGA-positive puncta were multivesicular body-like structures.

### 2.4 Volume in-resin CLEM of mitochondria in the Epon-embedded cells was performed by focused ion beam scanning electron microscopy

**Fig. 4:**
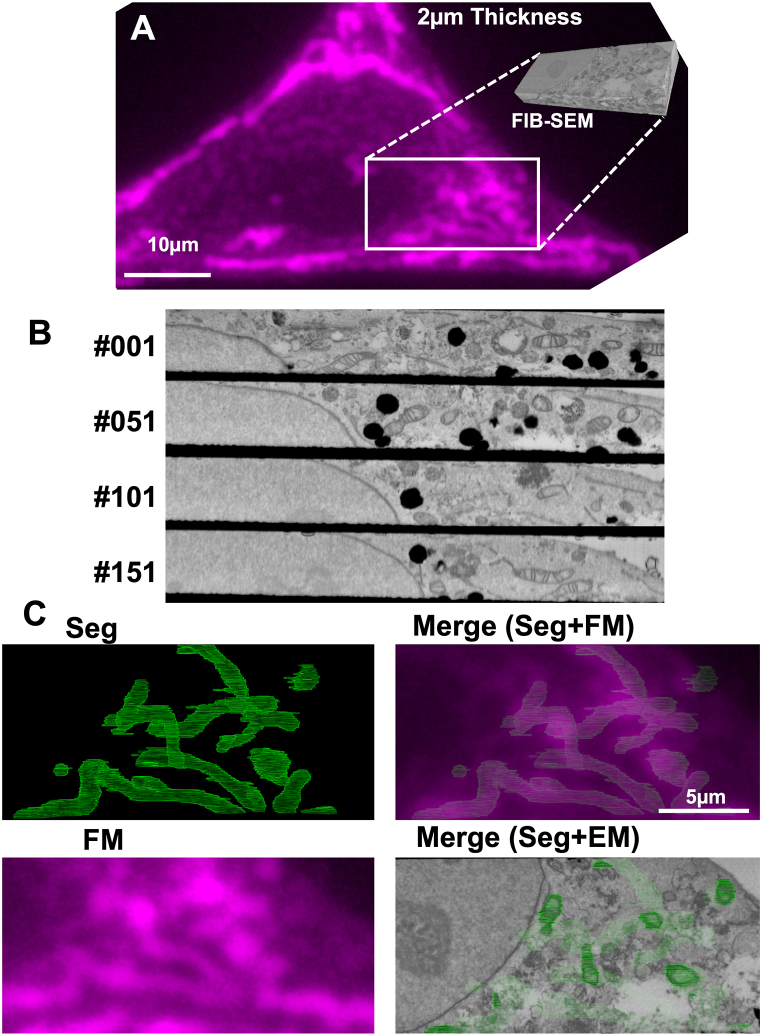
**Volume in-resin CLEM of mitochondria in the Epon-embedded HeLa cells was performed.** HeLa cells were incubated with anti-TOM20 antibody and Alexa Flour 555-streptavidin. After osmium tetroxide-staining and dehydration, cells were embedded in Epon-resins. Semithin sections (2 μm thick) were prepared using a microtome with a glass knife. (**A**) TOM20-positive fluorescence in the X-Y plane of the semithin section was observed with a BZ-X810 fluorescence microscope (CCD monochrome camera, Nikon CFI Plan Apochromat Lambda 100x Oil lens, gain +16 dB) using a TRITC filter set (excitation: 520–570 nm, dichroic mirror: 565 nm long pass, emission: 535–675 nm) (magenta pseudo color). Every serial electron microscopic image (20 nm thickness, total 240 images) in the X-Z plane was observed with a Helios NanoLab 660 FIB-SEM (a backscattered electron detector at an acceleration voltage of 2.0 kV and a current of 0.4 nA with a pixel dwell time of 30 μs), and reconstituted to a 3D image using an Amira software (Supplementary movie). (**B**) Out of 240 electron microscopic images, the **1**st, **51**st, **101**th, and **151**th images were shown. (**C**) Mitochondria were segmented from the serial SEM images (green pseudo color) using Amira software, and the segmented mitochondria in all images were reconstituted to a 3D image using Amira software. The X-Y plane of the 3D image of segmented mitochondria was shown in “**Segmentation**”. “**FM**” indicated a TOM20-positive fluorescent image in the same area of 2 μm Epon-embedded samples (magenta pseudo color). “**Merge (FM + Seg)**” indicated the merged image of “**Segmentation**” and “**FM**”. “**Merge (EM + Seg)**” indicated the merged image of “**Segmentation**” and the surface image of the X-Y plane of 3D reconstituted image of SEM.

In-resin CLEM using affinity-based labeling with fluorescent dyes has high positional accuracy in the correlation between fluorescent and electron microscopic images of the same thin section in Epon-embedded samples. Because of autofluorescence of Epoxy resins, fluorescence microscopy of Epon-embedded samples has rarely been performed. If the fluorescent signals of a fluorescent dye in thick sections (several μm thick) of the Epon-embedded cells are detected by fluorescence microscopy, volume in-resin CLEM of Epon-embedded cells will be achieved via a combination of FIB-SEM and fluorescence microscopy. We therefore investigated whether the Epon-embedded samples in which mitochondria were applied to volume in-resin CLEM using a FIB-SEM (Fig. 4). HeLa cells were fixed in the presence of glutaraldehyde, permeabilized with 50 μg/ml digitonin and 0.02% Triton X-100, and incubated with rabbit polyclonal anti-TOM20 antibody. Cells were further incubated with biotinylated anti-rabbit IgGs antibody and Alexa555-conjugated streptavidin. After osmium tetroxide-staining and dehydration with ethanol, cells were embedded in Epon-resins. We prepared semithin sections (2 μm thick) of Epon-embedded cells. Fluorescence derived from the anti-TOM20 antibody was well observed even in the 2 μm thick section showing mitochondria-like profiles and localization patterns in the X-Y plane by fluorescence microscopy (Fig. 4C, **FM**). We next investigated the intracellular ultrastructures of cells in the same 2 μm thick section in the X-Z plane by a Helios NanoLab 660 FIB-SEM. Serial electron microscopic images obtained by an FIB-SEM indicated that membrane structures including mitochondria and nuclear membrane were well preserved (Fig. 4B). Ultrastructures of mitochondria in the obtained serial electron microscopic images were segmented, and both the segmented mitochondria and electron microscopic images were reconstituted in three-dimension (3D) (Fig. 4C) (Supplementary movie). Fluorescent images of mitochondria in the 2 μm thick section of Epon-embedded HeLa cells were well correlated with the X-Y plane of the 3D mitochondria-segmented image, while this was partially correlated with the X-Y plane of the 3D SEM images (Fig. 4C, Segmentation vs FM, merge (Seg & FM)). These results indicated that volume in-resin CLEM of Epon-embedded cells was easily achieved when mitochondria were immunologically detected using the anti-TOM20 antibody with a fluorescent dye.

## 3 Discussion

Here we performed two-color in-resin CLEM of Epon-embedded samples with the application of immunological reaction and affinity-labeling using orange and far-red fluorescent dyes. Each fluorescent dye examined had enough fluorescent intensity after dehydration with ethanol following fixation in the presence of glutaraldehyde and osmium-tetroxide staining. Application of immunological reaction with fluorescent dyes (HiLyte 555 and iFlour 546) to in-resin CLEM of Epon-embedded samples revealed that in-resin CLEM of mitochondria and the Golgi apparatus in the Epon-embedded samples were easily performed only by the detection of endogenous proteins using immunological reactions and fluorescent dyes. In addition to immunological reactions with an orange fluorescence dye, we employed a lectin-based affinity-labeling with a far-red fluorescent dye and achieved two-color in-resin CLEM of Epon-embedded samples. These results suggested that as far as fluorescent signals were obtained, in-resin CLEM of organelles in Epon-embedded samples is easily performed by detecting endogenous targets with fluorescent dyes. Additionally, we identified intracellular WGA-positive puncta were multivesicular body-like structures. Furthermore, using high positional accuracy, we performed volume in-resin CLEM of 2 μm section of Epon-embedded cells.

For this in-resin CLEM, cells were fixed with 4% paraformaldehyde and 0.25% glutaraldehyde. Under these fixation conditions, intracellular ultrastructures were well preserved. Under lower concentrations of glutaraldehyde (for example 0.025–0.1%) in the presence of 4% paraformaldehyde, these ultrastructures were hardly preserved well. For the permeabilization of the fixed cells, we employed 50 μm/ml digitonin and 0.02% Triton X-100. Treatment of the fixed cells with higher concentrations of Triton X-100 inhibited the preservation of intracellular ultrastructures of organelles in the Epon-embedded cells. Therefore, it will be required to investigate the affinity-labeling conditions suitable for in-resin CLEM (for example higher concentration of antibody, longer incubation time, and temperature).

We also found that intracellular WGA-positive signals seem to be late endosome-like structures such as multivesicular bodies in HeLa cells. WGA-positive signals were detected as cytoplasmic puncta and plasma membrane in HeLa cells, while WGA is generally said to be localized to post-Golgi structures. At least punctate, not ring-shaped, WGA-signals in ultrathin sections of Epon-embedded cells are defined as multivesicular body-like structures. Multivesicular bodies have intracellular unique membranous structures. These unique ultrastructures of WGA-positive multivesicular body-like structures in addition to mitochondria are suitable for potentially another positional marker of in-resin CLEM, since positional marker(s) are indispensable to correlate unknown fluorescent signals in fluorescence microscopy with ultrastructures in electron microscopy in the wide field area.

There are some weak points to be improved in this in-resin CLEM of Epon-embedded samples with the application of immunological reaction and affinity-labeling using orange and far-red fluorescent dyes. To detect the endogenous target(s) in the Epon embedded cells by immunological reaction and affinity-labeling using fluorescent dyes, there are limited resolution and contrast in the fluorescent images, resulting in the large resolution gap between fluorescent and electron microscopic images. Therefore, at present, this in-resin CLEM method may be suitable for analyzing ultrastructures of at least a minimum of several hundred nanometers in diameter, such as mitochondria and multivesicular-like structures. However, this in-resin CLEM of Epon-embedded samples can analyze endogenous targets without recombinant techniques. This is a potentially great advantage in the analysis of non-recombinant cells and tissues, especially for the future study of diseased mammalian tissues. Based on this in-resin CLEM, we are now analyzing glial cells and their intracellular lysosomes and p62 aggregates in a mouse brain model of neurodegenerative disease. To apply this in-resin CLEM to mammalian tissues with better resolution and contrast, it is necessary to find more brighter fluorescent dyes and/or new resins with low autofluorescence while preserving intracellular ultrastructures and improving fluorescence microscopy.

Volume in-resin CLEM of mitochondria in 2 μm thick section of Epon-embedded cells with immunological reactions was performed. It is interesting that fluorescent images can be detected from 2 μm thick section of Epon-embedded cells, while Epon-resins have autofluorescence. If three-dimensional in-resin CLEM (about 2 μm thick) is performed using a series of serial sections of ultrathin sections (50∼100 nm thick), we need to obtain about 20–40 fluorescent and electron microscopic images, correlate all fluorescent and electro microscopic images, and stack them all. However, a single examination of 2 μm semi-thin section of Epon-embedded cells by fluorescence microscopy and FIB-SEM resulted in this volume in-resin CLEM. This volume in-resin CLEM is a potentially great advantage when performing CLEM of a larger area, such as mammalian tissues in the future.

We employed orange and far-red fluorescent dyes for two-color in-resin CLEM. For multi-color in-resin CLEM, more color fluorescent dyes will be required. We have examined the resistance of “green” fluorescent dyes including Alexa Fluor 488, DyLight 488, iFluor 488, and HiLyte 488 against osmium tetroxide-staining and dehydration with ethanol, and found that each fluorescent dye showed decreased but enough fluorescent intensity. However, probably because of autofluorescence of Epoxy resins, “green” fluorescence in Epon-embedded cells labeled with biotinylated WGA and fluorescent dye-conjugated streptavidin was hardly detected by fluorescence microscopy. It will be possible that “green” fluorescent dye is available for in-resin CLEM of Epon-embedded cells if the fluorescent intensity of “green” fluorescence is high enough to overcome autofluorescence of Epoxy resins. Brighter “green” fluorescent dye for the in-resin CLEM of Epon-embedded cells or autofluorescence-free epoxy-resins suitable for electron microscopy of biological materials will be awaited.

## 4 Materials and methods

### 4.1 Cells, media, and materials

HeLa cells were obtained from the American Type Culture Collection and were cultured in Dulbecco’s Modified Eagle’s Medium (DMEM, Nacalai Tesque, #08458-45) containing 10% fetal bovine serum (Equitech Bio, #268-1). Rabbit polyclonal anti-TOM20 antibody was purchased from Santa Cruz Biotechnology (#sc-11415), and mouse monoclonal anti-GM130 antibody was from BD Biosciences (#610822). Alexa Fluor 555 conjugated anti-mouse IgG (#A31570), Alexa Fluor 555 conjugated anti-rabbit IgG (#A31572), Alexa Fluor 555 conjugated streptavidin (#S21381), Alexa Fluor 647 conjugated streptavidin (#S21374), and Alexa Fluor 647 conjugated wheat germ agglutinin (WGA) (#W32466) were from Thermo Fisher Scientific. Biotinylated WGA (#B-1025) was from Vector Laboratories. DyLight 549 (#S000-42) and DyLight 649 (#S000-43) conjugated streptavidins were from Rockland. HiLyte Fluor 555 (#AS-60666) and HiLyte Fluor 647 (#AS-60667) conjugated streptavidins were from Anaspec. iFluor 555 (#16989) and iFluor 647 (#16996) conjugated streptavidins were from AAT Bioquest. ATTO 647 (#AD647-61) streptavidin was from ATTO-TEC. SPICA dye-conjugated streptavidin was from Fujifilm Wako Chemicals. Biotin-SP AffiniPure goat anti-rabbit IgG (#111-065-144) was purchased from Jackson ImmunoResearch.

### 4.2 Sample preparation, fluorescence microscopy, and electron microscopy

Cells were prefixed with a fixation solution containing 4% paraformaldehyde and 0.25% glutaraldehyde at 4 °C for 30 min [15]. The fixed cells were washed three times with HB solution (Fujifilm Wako Chemicals, # 080-10591). For WGA-staining, the cells were permeabilized with HB containing 50 μg/ml digitonin and 0.02% Triton-X100 at room temperature for 10 min and were incubated in a blocking solution (HB containing 1% bovine serum albumin (BSA)). Cells were incubated in an Abs solution (HB solution containing 0.1% BSA) with 1 μg/ml of biotinylated WGA (or Alexa Fluor 647-conjugated WGA) (1:1000) at room temperature for 60 min after washing the cells with HB solution three times. The cells were further incubated in an Abs solution containing fluorescent dye-conjugated streptavidin (1:1000) at room temperature for 30 min. For the application of immunological reactions for in-resin CLEM, cells were incubated in the Abs solution containing with anti-TOM20 (1:200) and anti-GM310 (1:100) antibodies at 37 °C for 120 min after permeabilization with 50 μg/ml digitonin and 0.02% Triton-X100 and were washed with HB solution at 37 °C for 10 min three times. They were further incubated in the Abs solution containing with biotinylated anti-rabbit IgG and anti-mouse IgG respectively at 37 °C for 120 min and were washed with HB solution at 37 °C for 10 min three times. Cells were further incubated in the Abs solution containing 1 μg/ml of fluorescent dye-conjugated streptavidin at room temperature for 30 min.

For embedding cells in Epon812 (Oken Shoji) resins, cells were post-fixed in 2% osmium tetroxide at 4 °C for 30 min, and washed three times with HB solution at 4 °C for 10 min. Cells were dehydrated with a graded series of ethanol and embedded in Epon812 at 60 °C for 72 h. Ultrathin sections (100 nm) were cut with an Ultramicrotome UC6 (Leica) and placed on 12-mm circular glass cover slips for scanning electron microscopy or TEM grids for transmission electron microscopy. The cover slips were pre-coated with Pt/Au (about 2.5-nm thickness) using an Ion Sputter E-1010 (Hitachi) to prevent the undesirable accumulation of negative charge from the incident electron beam on the surface of non-conductive specimens during scanning electron microscopy. Fluorescence images of sections were captured using a BZ-X810 fluorescence microscope (Keyence) with a CFI Plan Apochromat Lambda 100x Oil lens (Nikon) or a Nikon A1RHD25 confocal laser-scanning microscope with a CFI Plan Apochromat Lambda 60x lens (Nikon). The CFI Plan Apochromat Lambda lenses offered the highest level of chromatic aberration correction, resolution, and image flatness. By using these lenses, we confirmed that chromatic aberration is extremely low in orange to near red before observation with a fluorescence microscope. Thereafter the thin sections were observed via a Helios NanoLab 660 focused ion beam scanning electron microscope (FIB-SEM) (FEI), a Regulus 8240 scanning electron microscope (SEM) (HITACHI), and an HT7700 (HITACHI) transmission electron microscope (TEM).

When acquiring fluorescent images, we simultaneously captured images exposed appropriately to detect the desired organelle and images overexposed to reveal multiple whole cells and nuclei. For electron microscopic imaging, several corresponding cells were captured at low magnification. Based on the corresponding fluorescent images, we imaged the region corresponding to the desired fluorescence signals by electron microscopy, gradually increasing the magnification. The fluorescent and electron microscopic images were then correlated to establish matches between multiple cells and nuclei. Subsequently, the fluorescence images of intracellular organelles were correlated with electron microscopic images at a higher magnification. This involved enlarging the fluorescence images while aligning the more magnified electron microscopic images with the lower magnification electron microscopic images. Simultaneously, the position of cell membranes and the nuclear membrane, as determined from the overexposed fluorescence image, were correlated to identify their locations. Based on these techniques, we observed a strong correlation between the orange fluorescent signals of WGA and the multivesicular body-like structures as shown in Fig. 3. In addition, the far-red fluorescent signals of Tom20 were also found to correlate well with mitochondria (Fig. 3).

### 4.3 Volume in-resin CLEM

Semithin sections (2 μm thick) of Epon-embedded samples were prepared using an ultramicrotome with a glass knife and placed on a 12-mm circular glass cover slip. Fluorescent images were obtained using a BZ-X810 fluorescence microscope with a TRITC filter set. Thereafter the thin section was observed via a Helios NanoLab 660 FIB-SEM. Serial imaging was performed every 20 nm by using a backscattered electron detector (MD detector) at an acceleration voltage of 2.0 kV and a current of 0.4 nA with a pixel dwell time of 30 μs. The serial block-face FIB-SEM image of z-stack images was manually correlated using Amira 3D software (FEI). The 3D reconstruction was also performed with Amira.

### 4.4 Quantification of the fluorescent images and statistical analysis

For quantification of fluorescent cells were quantified using Image J software (https://imagej.nih.gov/ij/index.html). Statistical significances were evaluated by unpaired *t*-test. A *p*-value <0.01 was considered as reaching statistical significance.

## Author contribution statement

Isei Tanida: Conceived and designed the experiments; Performed the experiments; Analyzed and interpreted the data; Contributed reagents, materials, analysis tools or data; Wrote the paper.

Junji Yamaguchi, Chigure Suzuki, Soichiro Kakuta: Performed the experiments; Analyzed and interpreted the data; Contributed reagents, materials, analysis tools or data.

Yasuo Uchiyama: Conceived and designed the experiments; Wrote the paper.

## Funding statement

Yasuo Uchiyama was supported by Japan Agency for Medical Research and Development {21gm5010003}, MEXT-supported Program for the Strategic Research Foundation at Private Universities, Special Research in Subsidies for ordinary expenses of private schools from The Promotion and Mutual Aid Corporation for Private Schools of Japan, The Center of Genomic and Regeneration Medicine, Juntendo University Graduate School of Medicine.

Dr Isei Tanida was supported by Japan Agency for Medical Research and Development {22gm1710001 s0201}, Japan Society for the Promotion of Science {21H02435, 22H02872, 22H04652}, The Center of Genomic and Regeneration Medicine, Juntendo University Graduate School of Medicine.

Junji Yamaguchi was supported by Japan Society for the Promotion of Science {20K22744, 21K15198}.

Chigure Suzuki was supported by Japan Society for the Promotion of Science {22K07376}, The Research Institute for Diseases of Old Age, Juntendo University School of Medicine.

## Data availability statement

No data was used for the research described in the article.

## Declaration of competing interest

The authors declare that they have no known competing financial interests or personal relationships that could have appeared to influence the work reported in this paper.
